# Interprofessional education in the care of people diagnosed with dementia: protocol for a systematic review

**DOI:** 10.1136/bmjopen-2014-007490

**Published:** 2015-04-23

**Authors:** Ferruccio Pelone, Scott Reeves, Andreas Ioannides, Claire Emery, Kumud Titmarsh, Marcus Jackson, Anne Marie Hassenkamp, Nan Greenwood

**Affiliations:** Faculty of Health, Social Care and Education, Kingston University and St Georges, University of London, London, UK

**Keywords:** Alzheimer Disease, Education, Professional, Systematic review.

## Abstract

**Introduction:**

Interprofessional education (IPE) offers a possible way to improve interprofessional collaboration and patient care. Current research addressing the effectiveness of IPE in dementia care is limited. A protocol is described for a systematic review to investigate the evidence for the influence of IPE on collaborative knowledge and skills; interprofessional practice and the delivery of dementia care.

**Methods and analysis:**

We will search the following electronic databases: PubMed, EMBASE, The Cochrane Library, PsycINFO CINAHL, Applied Social Sciences Index and Abstracts (ASSIA), ERIC British Education Index (BEI) and the Healthcare Management Information Consortium (HMIC). Additional studies will be identified by manually searching relevant journals and the reference list of selected studies. The selection of the studies, data collection and quality appraisal will be performed independently by two reviewers. Data will be initially analysed through a narrative synthesis method. If a subset of data we analyse appears comparable, we will investigate the possibility of pooling such data via formal meta-analysis analytical techniques.

**Ethics and dissemination:**

Ethics approval will not be required as this is a protocol for a systematic review. This systematic review aims to establish the effectiveness of IPE programmes on collaborative professional practice and the delivery of care for people with dementia. The findings of this systematic review may also identify specific gaps in the evidence informing a future agenda for research, policy and practice. It will be published in a peer-reviewed journal.

**Trial registration number:**

PROSPERO CRD42014015075.

**Strengths and limitations of this study**

- Since we followed reliable and commonly used/well-tested methods to design this systematic review, our findings are likely to provide a comprehensive view of how interprofessional education is being used in dementia care–comparable with other scientific work.
- We will include a number of study designs including qualitative and quantitative research in order to provide information to help understand how these interventions may or may not be effective.
- Our systematic review is likely to have several limitations: (1) this is a comprehensive review of the current literature on interprofessional education and dementia care. However, it was limited to studies published in English; furthermore, literature on interprofessional education in dementia care might be limited and heterogeneous. Therefore, confounding and selection bias may occur; (2) to provide a current state of evidence, only studies published in the past 10 years were included in this review.

## Introduction

Given current epidemiological patterns,^1^ and as the diagnosis of dementia improves, the number of young and older adults diagnosed and treated for dementia-related conditions is projected to increase significantly.^2^ Dementia diagnosis, treatment and carers’ education are complex processes best achieved through collaboration among healthcare professions. Interprofessional education (IPE) offers a possible way to improve interprofessional collaboration and patient care.

Interprofessional education (IPE) has been defined as an activity that occurs when members of two or more professions (or students) learn with, from and about one another to improve collaboration and the quality of care.^3^ Earlier reviews have indicated that IPE can improve professional practice and health and social care outcomes in several fields such as child protection,^3^ delirium care^4^ diabetes care and domestic violence management.^5^

There is also some evidence for the potential effectiveness of IPE to improve collaborative knowledge and skills, interprofessional practice and the delivery of dementia care. For example, in a recent review Brody and Galvin^6^ found that interprofessional dementia education improved knowledge and attitudes for qualified staff, and was considered likely to improve outcomes. However, studies focused on IPE programmes involving students and unlicensed professions (eg, nursing assistants) were not included. In addition studies were excluded if they focused on forms of dementia other than Alzheimer's, Lewy body, vascular, mixed or frontotemporal.^6^

Therefore, the present systematic review aims to add to the ongoing development of evidence for IPE in dementia care, with potentially relevant implications for policymakers, researchers and professionals involved in supporting people with dementias and their carers. The review will be based on the following specific questions:

What is the evidence that IPE for providers involved in dementia care has benefits for (a) patients’ (health) outcomes; (b) family carers’(or caregivers as they are also known) outcomes; (c) providers’ (including students) education; (d) organisational and delivery of dementia care?
Does the implementation of IPE improve collaborative knowledge, skills and interprofessional practice (eg, in terms of educational outcomes and practice)?
(a) What are the outcomes of IPE in dementia care? (b) How does the context influence the achievement of the outcomes?

## Methods and analysis

### Criteria for considering studies for this review

#### Inclusion criteria

##### Types of participants

- Health and social care providers—regulated and unregulated—involved in caring for people with dementia.
- Students involved in caring for people with dementia.

##### Types of interventions

IPE is defined as an activity that occurs when members or students of two or more professions (health and social care providers—regulated and unregulated care providers) learn interactively (with, from and about) one another to improve collaboration and the quality of care. Any IPE intervention—including classroom-based, practice-based, simulation and online IPE activities at both the prelicensure and postlicensure qualification education levels—either delivered alone or in combination with other interventions will be included.

##### Type of studies

Both quantitative and qualitative research designs will be included in the review. The studies included in the final review are likely to include:

- Randomised studies (including randomised Clinical Trials (RCTs) and cluster RCTs);
- Non-randomised controlled studies (including controlled clinical trials; controlled before-after and interrupted time series studies);
- Cohort Studies (including follow-up studies; longitudinal studies; prospective studies; and retrospective studies);
- Qualitative studies (including phenomenological studies; ethnographic studies; grounded theory studies; and action research studies);
- Mixed method studies.

##### Types of outcome measures

**Figure 1:**
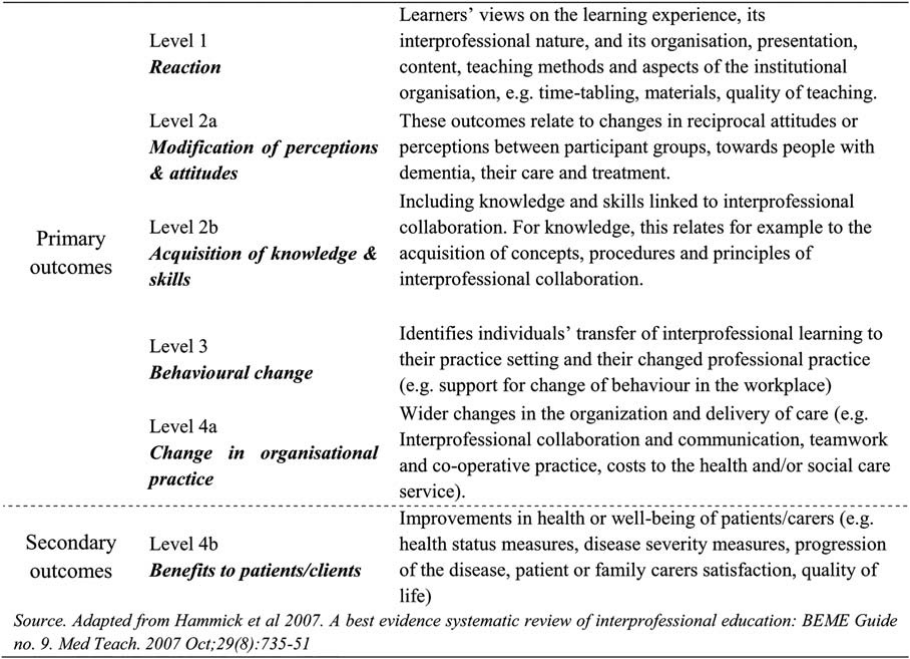
Modified Kirkpatrick's model to classify interprofessional education (IPE) outcomes.

A model will be used to classify the outcomes reported in each included paper (see figure 1). The proposed model seeks evidence in relation to six levels of outcomes.^3^

Primary outcomes: Reported educational and/or organisational outcomes for professions—including acquisition of collaborative knowledge and skills related to dementia care and changes in service delivery (eg, organisational practice, effectiveness, efficiency).

Secondary outcomes: These will focus on any reported changes in healthcare outcomes for people with dementia (eg, quality of life, well-being, mortality/morbidity and disease progression; level 4b in the outcomes typology).

#### Exclusion criteria

Articles will be excluded for the following reasons, even if they fulfil one or more inclusion criteria:

- Studies that do not involve people with dementia as described in the inclusion criteria;
- Studies that do not evaluate any intervention as described in the inclusion criteria;
- Studies that do not report empirical findings;
- Studies that do not report objectively measured patient/staff or healthcare process outcomes;
- Systematic reviews, commentaries and non-peer-review articles. Furthermore, unpublished literature will be excluded from the review although they will be used as sources of potentially relevant studies.

### Search methods for identification of studies

Relevant literature will be identified from systematic searches of electronic databases, manual searching and reference checking.

#### Electronic searches

- PubMed—table 1
- EMBASE—online supplementary file 1;
- The Cochrane Library—online supplementary file 1;
- PsycINFO—online supplementary file 1;
- CINAHL—online supplementary file 1;
- Applied Social Sciences Index and Abstracts (ASSIA)—online supplementary file 1;
- ERIC—online supplementary file 1;
- British Education Index (BEI)—online supplementary file 1;
- Healthcare Management Information Consortium (HMIC)—online supplementary file 1.

A PubMed search strategy has been developed according to the search questions, as well as the inclusion/exclusion criteria (see table 1). This has been converted to run on other databases (see online supplementary file 1). Results of electronic databases’ searches will be limited to the past 10 years (from 2004 to 2014) and to articles written in English.

**Table 1:** MEDLINE (PubMed) search strategy: 2004–2014, searched on 9 Sept 2014

Search #	Concept	Search String	Hits
1	Interprofessional	((((((((((((((((((((((((((((team*[Title/Abstract]) OR multiprofession*[Title/Abstract]) OR multi-profession*[Title/Abstract]) OR multi profession*[Title/Abstract]) OR multidisciplin*[Title/Abstract]) OR multi-disciplin*[Title/Abstract]) OR multi disciplin*[Title/Abstract]) OR multinstitution*[Title/Abstract]) OR multi-institution*[Title/Abstract]) OR multi institution*[Title/Abstract]) OR multioccupation*[Title/Abstract]) OR multi-occupation*[Title/Abstract]) OR multi occupation*[Title/Abstract]) OR multiorganization*[Title/Abstract]) OR multi-organization*[Title/Abstract]) OR multi organization*[Title/Abstract]) OR multiorganisation*[Title/Abstract]) OR multi-organisation*[Title/Abstract]) OR multi organisation*[Title/Abstract]) OR transprofession*[Title/Abstract]) OR trans-profession*[Title/Abstract]) OR trans profession*[Title/Abstract]) OR transdisciplin*[Title/Abstract]) OR trans-disciplin*[Title/Abstract]) OR trans disciplin*[Title/Abstract])) OR ((((((((((((((((((((((((interprofession*[Title/Abstract]) OR inter-profession*[Title/Abstract]) OR inter profession*[Title/Abstract]) OR interdisciplin*[Title/Abstract]) OR inter-disciplin*[Title/Abstract]) OR inter disciplin*[Title/Abstract]) OR interinstitut*[Title/Abstract]) OR inter-institut*[Title/Abstract]) OR inter institut*[Title/Abstract]) OR interagen*[Title/Abstract]) OR inter-agen*[Title/Abstract]) OR inter agen*[Title/Abstract]) OR intersector*[Title/Abstract]) OR inter-sector*[Title/Abstract]) OR inter sector*[Title/Abstract]) OR interdepartment*[Title/Abstract]) OR inter-department*[Title/Abstract]) OR inter department*[Title/Abstract]) OR interorganization*[Title/Abstract]) OR inter-organization*[Title/Abstract]) OR inter organization*[Title/Abstract]) OR interorganisation*[Title/Abstract]) OR inter-organisation*[Title/Abstract]) OR inter organisation*[Title/Abstract])))	317 809
		OR	
		(((Interdisciplinary Communication[MeSH Terms]) OR professional-patient relations[MeSH Terms]) OR interprofessional Relations[MeSH Terms])	
2	Education	(((education, continuing[MeSH Terms]) OR education, graduate[MeSH Terms])) OR (((((education*[Title/Abstract]) OR trainin*[Title/Abstract]) OR learn*[Title/Abstract]) OR teach*[Title/Abstract]) OR course*[Title/Abstract])	1 287 500
3	Interprofessional education	#1 AND 2	68 046
4	Dementia	(dement*[Title/Abstract]) OR (((((dementia[MeSH Terms]) OR Alzheimer Disease[MeSH Terms]) OR Frontotemporal Dementia[MeSH Terms]) OR Lewy Body Disease[MeSH Terms]) OR Dementia, Vascular[MeSH Terms])	142 026
5	Interprofessional education publications in dementia excluding editorials (published in the last 10 years; English)	#3 AND 4	651
6		((comment[Publication Type]) OR letter[Publication Type]) OR editorial[Publication Type]	1 342 938
7		#5 NOT #6	645
8		#7 Filters: published in the last 10 years, Humans, English	303

#### Other sources

Additional studies will be identified:

- Manual searching of three relevant journals (past 10 years; *Journal of Interprofessional Care*; *International Psychogeriatrics; Dementia*: *The International Journal of Social Research and Practice*). These journals have the highest numbers of published papers on IPE and dementia;
- Checking the reference lists of included studies;
- Checking the reference lists of pertinent studies;
- Consulting corresponding authors of key studies to identify any other relevant article.

### Data collection

#### Selection of studies

The screening process will be conducted in three stages: (1) duplicate removal, (2) screening titles/abstracts; and (3) full-texts screening. A check-list will be developed to guide the screening process at each stage (see online supplementary file 2). The second and third stage of the selection process will start with a pilot, aimed at ensuring the consistency among reviewers in applying the eligibility criteria.

##### Duplicate citations

The results of the literature search will be downloaded into an Excel spreadsheet. Article duplicates will be removed. Multiple publications from the same study population identified during full-text review will be screened for duplication of data.

##### Title and abstract screening

Two reviewers will independently screen all titles and abstracts. Any discrepancies will be resolved by a third reviewer. A copy of articles that appear to meet the inclusion criteria based on the title and abstract screen will be obtained for full-text review. Full-text copies of articles where it cannot be determined whether it is relevant on the basis of the title and abstract will also be obtained to determine eligibility based on full-text review.

##### Full-text screening

Full-paper manuscripts of any relevant titles/abstracts will be obtained where possible and will be independently scrutinised by two members of the review team with reasons for exclusion annotated and tracked. Any discrepancies will be resolved by a third reviewer. The primary reason for excluding studies will be if the article does not contain original data relevant to our eligibility criteria.

At this stage of the screening process, one member of the review team will independently scan the reference lists of the included studies and relevant reviews for references that were not identified from the database searches. Eligibility will be discussed with a second reviewer and the source of the citation tracked.

#### Data extraction and management

##### Data extraction

Two reviewers will independently extract data for each included paper. Data to be extracted will include: study design, participant characteristics, description of the intervention and study findings. A form for data extraction will be piloted on five studies to refine the checklist and ensure that the data extraction tool captures all of the intricacies of both qualitative and quantitative designs.

##### References and data management

**Figure 2:**
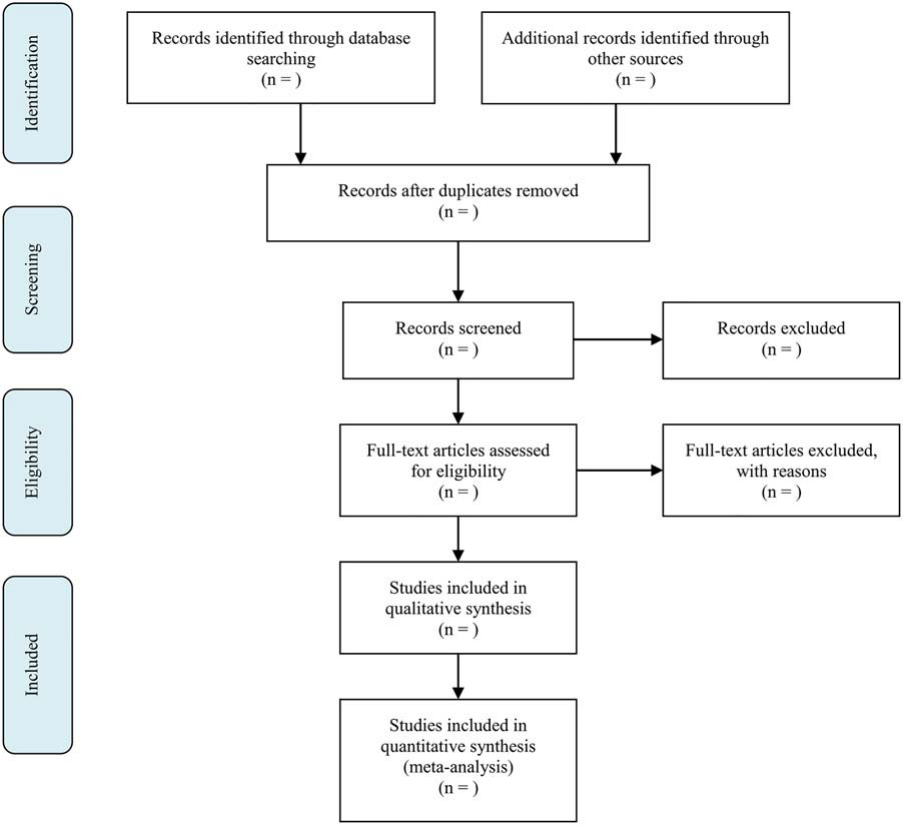
Flow diagram of systematic review.

Excluded studies with reason for exclusion, as well as of the overall selection process will be recorded by means of an Excel spreadsheet. Hard copies of titles, abstracts and full texts to be reviewed at any stage of the screening process will be printed out, while the reviewers’ assessments and their comments will be managed electronically via Excel spreadsheets. Study data will be extracted using standard forms and entered into Excel spreadsheets in tabular form. The flow of information through the different stages of the systematic review will be documented in a schematic (figure 2)—as recommended in the PRISMA statement on preferred reporting items for systematic reviews and meta-analyses.^7^

#### Assessment of risk of bias in included studies

Quality assessment will be undertaken independently by two members of the review team with discrepancies resolved by consensus or recourse to a third reviewer if necessary. There has been a long-standing controversy in the literature regarding critical appraisal of both quantitative and qualitative research studies due to the inherently different approaches taken to the collection, analysis and writing up of data. A search was conducted to identify the most suitable tools to assess the methodological quality of studies included in this review. Following this search, we selected the checklists for quantitative or qualitative studies of the Alberta Heritage Foundation for Medical Research.^8^ The broad nature of these quality assessment tools will allow a range of methodologies to be assessed. Both quantitative and qualitative studies will be scored depending on how fully they met all criteria (11 for quantitative studies, 10 for qualitative studies). If a criterion is not applicable, then it will be excluded from the score calculation. The proposed review will use the criteria recommended by the GRADE Working Group to assess the strength of the body of evidence across particular study outcomes.^9^

#### Strategy for data analysis

If a subset of data we analyse appears comparable, we will investigate the possibility of pooling such data via formal meta-analysis analytical techniques. Specifically, a random-effects model meta-analysis will be performed using by using RevMan 5.2.1, if there is no evidence of heterogeneity among studies in terms of design and measurement of outcomes.

Owing to the heterogeneity of the studies we expect to find, data will be initially analysed through a narrative synthesis method. For this purpose, the included studies will be grouped into outcome type using a modified Kirkpatrick's model to classify IPE outcomes (see figure 1–ie, reaction, modification of perceptions and attitudes, acquisition of knowledge and skills, behavioural change, change in organisational practice, benefits to people with dementia and/or their carers); then subgrouped by IPE intervention content using as guidance the 3-P (Presage, Process and Product) framework and finally for target population characteristics. The 3-P (presage, process and product) model—proposed by Biggs^10^ elaborated in the context of IPE by Freeth and Reeves,^11^ is a useful tool for describing and analysing IPE. Many reviews recently applied the 3-P model to the evaluation of IPE interventions.^3^ ^11^ ^12^ They found that the model was very useful to understand the links between factors that provide the context in which the learning experience is conducted, such as learners and teachers/facilitators’ characteristics (ie, presage) in relation to the delivery of IPE (ie, process) and the outcomes of learning (ie, product).

#### Ethics and dissemination

Ethics approval will not be required, since this is a protocol for a systematic review utilising published data. Once completed, the results from this systematic review will be published in a peer-reviewed journal.

## Implications

This systematic review aims to establish the effectiveness of IPE programmes on collaborative knowledge and skills, interprofessional practice and the delivery of dementia care. Evidence from other health conditions such as delirium^4^ suggests that IPE can enhance healthcare but the evidence for the impact of IPE on both training professionals in supporting neither people with dementia nor their carers has not been systematically explored. We will therefore examine the factors influencing on professionals involved in the care of people with dementia and their carers. Results of this systematic review will therefore provide new insights into education approaches to increase healthcare providers’ competence in caring for people with dementia, exploring the benefits of IPE in the field of dementia care. The systematic review may also identify specific gaps in the evidence, which would inform a future agenda for research, policy and practice.

### Amendments

If we need to amend this protocol, the date, rationale and a description of each protocol change will be reported.
